# Maternal Folic Acid Supplementation Improves the Intestinal Health of Offspring Porcine by Promoting the Proliferation and Differentiation of Intestinal Stem Cells

**DOI:** 10.3390/ani13193092

**Published:** 2023-10-03

**Authors:** Yuhui Zhang, Xiaofeng Zhang, Jianjun Chen, Shouchuan Jiang, Yu Han, Huahua Du

**Affiliations:** 1 Key Laboratory of Animal Nutrition and Feed Science (Eastern of China), College of Animal Sciences, Zhejiang University, Hangzhou 310058, China; 2 Institute of Animal Husbandry and Veterinary Science, Zhejiang Academy of Agricultural Sciences, Hangzhou 310004, China

**Keywords:** folic acid, folate, maternal nutrition, proliferation, differentiation, intestinal stem cells

## Abstract

**Simple Summary:**

Maternal folic acid intake is crucial for offspring growth and development. This study investigated the impact of maternal folic acid supplementation on the small intestine development in piglets. We found that folic acid supplementation during gestation and lactation significantly increased the body weight, villus length, and expression of nutrient transporters in the duodenum and jejunum of offspring piglets. Additionally, maternal folic acid supplementation also enhanced the proliferation and differentiation of intestinal stem cells of piglets. These results highlight the beneficial effects of maternal folic acid supplementation on growth performance and gut health of offspring by enhancing the balance of epithelial cell renewal.

**Abstract:**

Maternal folic acid intake has important effects on offspring growth and development. The mechanism involved in the renewal of intestinal epithelial cells remains unclear. Thus, this study aimed to investigate the potential effect of maternal folic acid supplementation during gestation and lactation on the structural and functional development of the small intestine in piglet offspring. Twenty-four Duroc sows were assigned to a control group (CON) and a folic-acid-supplemented group (CON + FA, supplemented with 15 mg/kg of folic acid). The results showed that maternal folic acid supplementation throughout gestation and lactation significantly increased the body weight, serum folate level, and intestinal folate metabolism in piglets. It also improved the villus length, villus height-to-crypt depth ratio, and transcript levels of nutrient transporters (GLUT4, SNAT2, FABP2, and SLC7A5) in piglets’ duodenum and jejunum. In addition, maternal folic acid supplementation increased Ki67-positive cells and the expression of proliferation-related marker genes (C-Myc, CyclinD1, and PCNA) in piglets’ intestinal stem cells. It also boosted the expression of genes associated with mature secreted cells (ChrA, Muc2, Lyz, Vil1), indicating enhanced proliferation and differentiation of intestinal stem cells. These findings demonstrate that maternal folic acid supplementation enhances growth performance and gut health in piglet offspring by promoting epithelial cell renewal equilibrium.

## 1. Introduction

The maternal environment is crucial for embryonic development and significantly influences the growth and development of infants [1]. Folic acid has been demonstrated to be an indispensable nutrient for the proper development of embryos and newborns [2]. In the developing fetus, maternal folic acid supplementation can change DNA methylation and gene expression, which may increase the risk of acquiring certain diseases in later life [3].

Folate, a naturally occurring derivative of folic acid, is an essential micronutrient that serves as a vital cofactor in one-carbon transfer reactions, nucleic acid synthesis, and biological methylation reactions [4]. Dietary folic acid contributes to the prevention of cardiovascular disease [5] and colon cancer [6], as well as to promoting the development of skeletal muscle [7]. It is important to note that folates cannot be synthesized within mammalian cells and must be obtained from dietary sources and the gut microbiota [8]. It has been demonstrated that undesirable pregnancy and birth outcomes including neural tube defects are linked to inadequate folate status during pregnancy [9]. Maternal folic acid intake throughout the preconception period and the initial four weeks of pregnancy has been found to significantly reduce the occurrence and recurrence of neural tube defects [10]. It is currently advised that women who are attempting to conceive or are planning a pregnancy should consume a daily supplement containing 400–800 mg of folic acid [11]. Maternal folic acid supplementation can enhance the intestinal function in offspring by improving intestinal one-carbon metabolism [12]. Hence, a growing body of evidence suggests that maternal folic acid supplementation may play an important role in the maturation and health of the neonatal gut.

The proper development of the gut in newborns, particularly in terms of cell renewal, is of utmost importance during the weaning process. The intestinal morphology and functions are negatively impacted by weaning stress, which causes villus atrophy, crypt hyperplasia, impaired barrier function, impaired cell renewal, and altered metabolism [13]. Notably, folic acid supplementation has been shown to improve the intestinal morphology of weaned piglets by preserving the equilibrium of epithelial cell renewal [14]. Folic acid plays a crucial role in DNA and RNA synthesis, gene expression regulation through epigenetic mechanisms, and the modulation of cell proliferation and differentiation [15]. Previous studies have highlighted the significance of folic acid in the growth of various cancer cells and adult cells, emphasizing its essentiality in the self-renewal and differentiation processes of specific adult stem cells [16,17]. However, limited knowledge exists regarding the impact of folic acid on the proliferation and differentiation of intestinal stem cells.

Existing studies have indicated a hypothesis concerning a potential inherent relationship between folic acid, intestinal stem cells, and gut health. Nevertheless, the precise impact of maternal folic acid supplementation on proliferation and differentiation of intestinal stem cells in offspring remains uncertain. Therefore, we carried out this study to explore the effect of maternal folic acid supplementation on the development of gut health, intestinal function, and the proliferation and differentiation of intestinal stem cells in offspring.

## 2. Materials and Methods

### 2.1. Experimental Design

All protocols of animal experiments were approved by the Institutional Animal Care and Use Committee of Zhejiang University (protocol code ZJU012302). A total of 24 Duroc sows (3–5 parities) with the same genetic background were selected and randomly divided into a control group (CON, *n* = 12) with basal diet and a CON + FA group (*n* = 12), supplemented with 15 mg/kg folic acid (Sigma-Aldrich, St. Louis, MI, USA). The basal level of folic acid was 1.3 mg/kg. The diets met the nutrient requirements established by the NRC (2012) and contained equal amounts of crude protein and digestive energy. The ingredients and nutrient levels of the basal diet are shown in Table 1. Sows were fed different diets from 30 days after the last artificial insemination (pregnancy was confirmed by ultrasonic examination 30 days post-mating) until the day the piglets were weaned. In accordance with the 2012 NRC recommendations and the usual breeding management of the pig farm, all sows were housed in groups during gestation, and the 12 experimental replicates within each group were fed individually. Subsequent to farrowing, lactating sows and their piglets cohabitated, with distinct groups of sows being, respectively, offered either the basal diet or a diet supplemented with folic acid ad libitum until the piglets reached the age of 35 days and were subsequently weaned. Blood samples were collected from sows via the precaval vein 100 days post-conception. Prior to suckling, the udder was carefully cleansed with water, and colostrum samples (30–40 mL) were manually extracted from the sows after parturition. The serum and colostrum samples were immediately placed in a −20 °C low-temperature freezer for future analysis. The body weight and survival rate of the piglets were monitored once a week. For tissue and blood collection, weaned piglets (5 piglets per group, their body weight being as close as possible to the average BW in each treatment group) were slaughtered through CO_2_ inhalation and exsanguination at the age of 35 days. Some tissue samples of duodenum, jejunum, and ileum were frozen in liquid nitrogen to prevent degradation and inactivation of biomolecules in the samples and stored at −80 °C for analysis. Other intestinal tissue samples were preserved with 4% paraformaldehyde to form intestinal slices and analyzed morphologically.

### 2.2. Serum and Colostrum Biochemical Assays

Folate analysis was conducted on serum samples obtained from weaning piglets, utilizing a folate reagent kit (Abbott Laboratories, Santry Dublin, Ireland). Similarly, homocysteine levels were assessed using a homocysteine reagent kit (Zybio, Chongqing, China), following the manufacturer’s instructions. Cytokine (IL-6, TNF-α), IgG, IgM, and diamine oxidase (DAO) concentrations in colostrum of sows and serum of weaning piglets were determined using ELISA kits (cat. No. MB-2558A, cat. No. MB-2523A, cat. No. MB-2543A, cat. No. MB-2542A, cat. No. MB-2578A, Meibiao Biological Technology, Yancheng, China) and quantified at 450 nm using a microplate reader (Bio-Rad, Shanghai, China). The ELISA kits for IL-6, TNF-α, IgG, IgM, and DAO concentrations exhibited a minimum detection limit of 25 pg/mL, 6.25 pg/mL, 0.75 mg/mL, 0.625 mg/mL, and 0.1 ng/mL, respectively.

### 2.3. Histological Analysis

Intestinal tissue samples were fixed in 4% paraformaldehyde, gradually dehydrated in ethanol (from low to high concentrations up to 100%), embedded in clear paraffin, and cut into 5 μm slices. For hematoxylin and eosin (H&E) staining, sections were deparaffinized with xylene and rehydrated with varied amounts of alcohol before being stained with hematoxylin for 5 min. At room temperature, the sections were distinguished in aqueous hydrochloric acid for 2 s and blunted in aqueous ammonia for 15–30 s. The tissue slices were then stained with eosin for 5–8 s. Following a gradual dehydration process involving ethanol and xylene, the sections were mounted using neutral gum. The resulting H&E staining was examined under a light microscope (Olympus, Tokyo, Japan). Crypt depth and villus length were measured using Image-Pro Plus 6.0 software (Media Cybernetics, Rockville, MD, USA). Each slice had at least six pairs of villus and crypt. For PAS staining, duodenum and jejunum slices were stained with periodic acid–Schiff (PAS) in accordance with industry standards to count the goblet cells. The number of goblet cells was counted in slices after a routine PAS staining by blindly counting the positive vacuoles. A DM3000 microscope (Leica, Wetzlar, Germany) was used to examine each slice.

### 2.4. Quantitative Real-Time PCR

Utilizing TRIzol reagent (TransGen Biotech, Beijing, China), total RNA was extracted from intestinal tissues. RNA quality was determined using a NanoDrop 2000 sectrophotometer (Thermo Fisher Scientific, Shanghai, China) and RNA was reverse-transcribed into cDNA using a RT Master Mix kit (Yeasen Biotech, Shanghai, China). Real-time qPCR was performed with quick-start universal SYBR green master (Yeasen Biotech, Shanghai, China) using the ABI 7500 Real Time PCR system (Thermo Fisher Scientific, Shanghai, China) and following the manufacturer’s guidelines. The melting curve analysis performed after the target gene amplification determined the specificity of the amplification. After *β-actin* reference was normalized, the relative mRNA expression of the target genes was calculated using the 2^−ΔΔCt^ method. Primer 5.0 software (Premier Biosoft, Palo Alto, CA, USA) was used to create the primer sequences, and BLAST sequence alignment in NCBI was used to confirm their validity (Table 2).

### 2.5. Western Blot Analysis

A total protein extraction kit (Solarbio Science & Technology, Beijing, China) was used to extract protein from the duodenum and jejunum tissues. The BCA protein assay kit (Biosharp, Beijing, China) was used to determine the amount of protein in samples. Protein extracts were separated via 10% SDS-PAGE gels and transferred to a polyvinylidene difluoride (PVDF) membrane (Millipore, Shanghai, China). The membranes were blocked with 5% skimmed milk and incubated with anti-FABP2 (1:1000; cat. No. ER1911-53, HUABIO, Hangzhou, China), anti-SLC7A5 (1:1000; cat. No. ER1916-84, HUABIO, Hangzhou, China), anti-PCNA (1:1000; cat. No. ET1605-38, HUABIO, Hangzhou, China), anti-ChrA (1:1000; cat. No. ab15160, Abcam, Waltham, MA, USA), anti-Vil1 (1:1000; cat. No. 16488-1-AP, Proteintech, Wuhan, China), and anti-β-actin (1:1000; cat. No. AC026, ABclonal, Woburn, MA, USA) antibodies at 4 °C overnight. After being washed, the membranes were incubated at room temperature for 1 h with HRP-linked goat anti-rabbit IgG (1:5000; cat. No. BL003A; Biosharp, Beijing, China). Finally, protein bands were detected using an enhanced chemiluminescence (ECL) assay kit (Biosharp, Beijing, China), quantified using Image J software (NIH, Bethesda, MD, USA), and adjusted to β-actin protein intensity.

### 2.6. Immunofluorescence

Porcine jejunal tissue slices were permeabilized for 10 min in 0.5% Triton X-100 in PBS (Servicebio, Wuhan, China) and incubated with blocking buffer (3% BSA and 0.3% Triton X-100 in PBS) for 1 h. Then, slices were incubated with a primary antibody against Ki67 (1:1000; cat. No. ab15580, Abcam, Waltham, MA, USA) at 4 °C overnight. After washing, the slices were incubated with a secondary antibody for 1 h at room temperature, and stained with nuclei for 8 min. An Olympus BX63 fluorescent microscope with a 20× objective (Olympus, Tokyo, Japan) was used to take single-channel grayscale pictures of the immunofluorescence staining.

### 2.7. Statistical Analysis

All animal performance and molecular biological data were presented as the mean ± standard error of the mean (SEM). The data were analyzed using two-tailed Student’s *t* test in GraphPad Prism 8.0 (GraphPad Software, USA). Statistical significance was defined as a *p* value of 0.05. The symbols for statistical significance are * *p* < 0.05 or ** *p* < 0.01.

## 3. Results

### 3.1. Maternal Folic Acid Supplementation Increased Offspring Growth Performance

**Figure 1:**
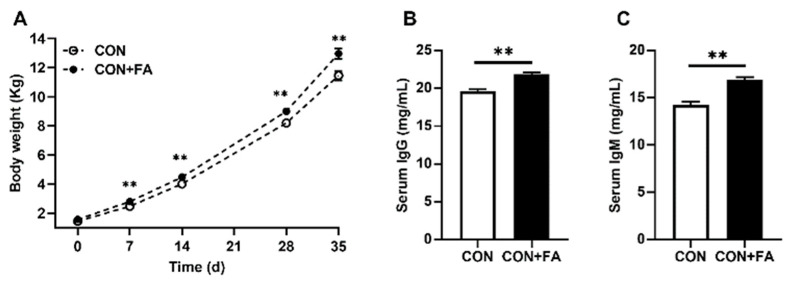
Maternal folic acid supplementation increased offspring growth performance. (**A**) Body weight of the piglets. (**B**) Serum IgG level. (**C**) Serum IgM level. The data are shown as means ± SEM of three independent tests. Tukey’s *t*-test between the two groups and one-way ANOVA among multiple groups were used to generate *p* values. ** *p* < 0.01 indicate a significant difference (*n* = 5).

No dams failed to acquire weight or lost weight, and no dams died prematurely. There were no statistically significant variations in the average number and birth weight of pups per litter between the two maternal diet groups. There was also no significant difference in the initial weight of newborn piglets between the CON and CON + FA groups (Figure 1A). However, the body weight was significantly (*p* < 0.01) increased at 7 d until 35 d in the piglets born to dams with folic acid supplementation. At a 35 d age, the average body weight of piglets was significantly (*p* < 0.01) higher in the CON + FA group (12.97 ± 0.35) compared with that in the CON group (11.46 ± 0.33). In addition, the concentration of serum immunoglobulin G (IgG) and IgM, two kinds of immunoglobulins commonly found in blood, were both significantly increased, by 12% (*p* < 0.01) and 19% (*p* < 0.01), respectively, in the piglets with maternal folic acid supplementation (Figure 1B,C). This suggested that maternal folic acid supplementation could increase the offspring’s growth performance and modulate its immunity.

### 3.2. Maternal Folic Acid Supplementation Increased Intestinal Folate Metabolism in Offspring

**Figure 2:**
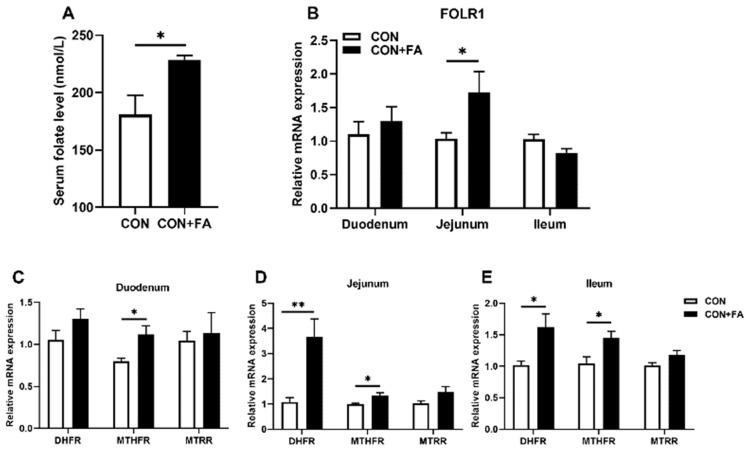
Maternal folic acid supplementation increased intestinal folate metabolism in offspring. (**A**) Serum folate level. (**B**) Relative mRNA expression level of *FOLR1* gene in the duodenum, jejunum, and ileum of piglets. (**C**–**E**) Relative mRNA expression levels of key enzymes of folate metabolism, *DHFR*, *MTHFR*, and *MTRR*, in the duodenum, jejunum, and ileum. The mRNA expression was normalized to *β-actin*. The data are shown as means ± SEM of three independent tests. Tukey’s *t*-test between two groups and one-way ANOVA among multiple groups were used to generate *p* values. * *p* < 0.05, ** *p* < 0.01 indicate a significant difference (*n* = 5).

In order to determine if the good growth performance of offspring was related to folic acid supplementation, we examined the gene expression of folate receptor FOLR1 and key enzymes in folate metabolism in the piglets. As expected, the serum folate concentration of piglets born to dams on the folic-acid-supplemented diets were significantly (*p* < 0.05) higher by 27% than that of the piglets born to dams on control diets (Figure 2A). The transcript of *FOLR1* was also notably (*p* < 0.05) increased in the jejunum of piglets born to dams with folic acid supplementation, while it remained no different in the duodenum and ileum (Figure 2B). Dihydrofolate reductase (DHFR), 5, 10 methylenetetrahydrofolate reductase (MTHFR), and methionine synthase reductase (MTRR) are three ubiquitous enzymes, which play essential roles in the folate metabolism pathway. The mRNA levels of *MTHFR* were all significantly (*p* < 0.05) higher in the duodenum, jejunum, and ileum of piglets born to dams with folic acid supplementation than those of piglets born to dams in the control group (Figure 2C–E). Meanwhile, the gene expressions of *DHFR* were significantly (*p* < 0.05) increased in the jejunum and ileum of piglets born to dams with folic acid supplementation, while *MTRR* remained unchanged. The above results indicated that maternal folic acid supplementation could increase the folate level of the offspring and promote its metabolism in the intestinal tract. 

### 3.3. Maternal Folic Acid Supplementation Enhanced the Integrity of Intestinal Morphology in Offspring

**Figure 3:**
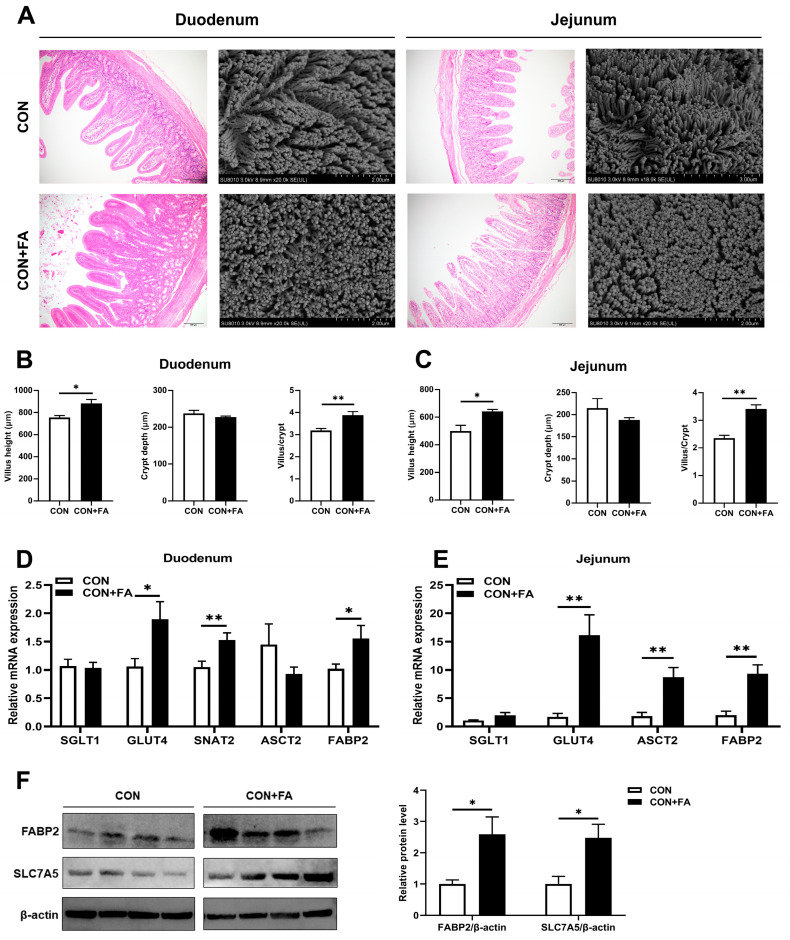
Maternal folic acid supplementation enhanced the integrity of intestinal morphology in offspring. (**A**) Morphology of duodenum and jejunum were analyzed via H&E staining (scale bar = 200 μm) and SEM observation (Scale bar = 2 μm). (**B**,**C**) Villus height and crypt depth of H&E images in the duodenum and jejunum. (**D**) Relative mRNA expression levels of nutrient transporter genes, *SGLT1*, *GLUT4*, *SNAT2*, *ASCT2*, and *FABP2*, in the duodenum. (**E**) Relative mRNA expression levels of nutrient transporter genes, *SGLT1*, *GLUT4*, *ASCT2*, and *FABP2*, in the jejunum. (**F**) Relative protein expression of FABP2 and SLC7A5 in the jejunum. The mRNA and protein expression were both normalized to *β-actin*. The data are shown as means ± SEM of three independent tests. Tukey’s *t*-test between two groups and one-way ANOVA among multiple groups were used to generate *p* values. * *p* < 0.05, ** *p* < 0.01 indicate a significant difference (*n* = 5).

H&E staining images of duodenum and jejunum tissues showed that the villus length in folic-acid-supplemented piglets was noticeably longer than that of control piglets (Figure 3A). SEM observation showed slight surface damage with uneven microvilli in the weaned control piglets, and folic acid supplementation restored the quality of microvilli to some extent (Figure 3A). Both in the duodenum and jejunum, the growth of villi in folic-acid-supplemented piglets was better, with significantly (*p* < 0.05) longer length and a significantly (*p* < 0.01) increased ratio of villus height to crypt depth (Figure 3B,C). Since the intestinal villus structure contributes to nutrient absorption, we then measured the expression of genes encoding the sugar transporters SGLT1 and GLUT4, amino acid transporters SNAT2, ASCT2, and SLC7A5, and fatty acid transporter FABP2 in the duodenum and jejunum of piglets. The transcripts of *GLUT4*, *SNAT2*, and *FABP2* were all significantly (*p* < 0.05) increased in the duodenum of piglets with folic acid supplementation (Figure 3D). And in the jejunum, the mRNA expressions of *GLUT4*, *ASCT2*, and *FABP2* were significantly (*p* < 0.01) higher in the piglets with folic acid supplementation compared with those in control piglets (Figure 3E). Furthermore, Western blot analysis confirmed that the protein levels of FABP2 and SLC7A5 were significantly increased in the jejunum of folic-acid-supplemented piglets compared with control piglets (Figure 3F). The results indicated that maternal folic acid supplementation could enhance the integrity of the intestinal morphology of offspring, which is beneficial to nutrient absorption of the intestinal mucosa.

### 3.4. Maternal Folic Acid Supplementation Improves Intestinal Inflammatory Response in Offspring

**Figure 4:**
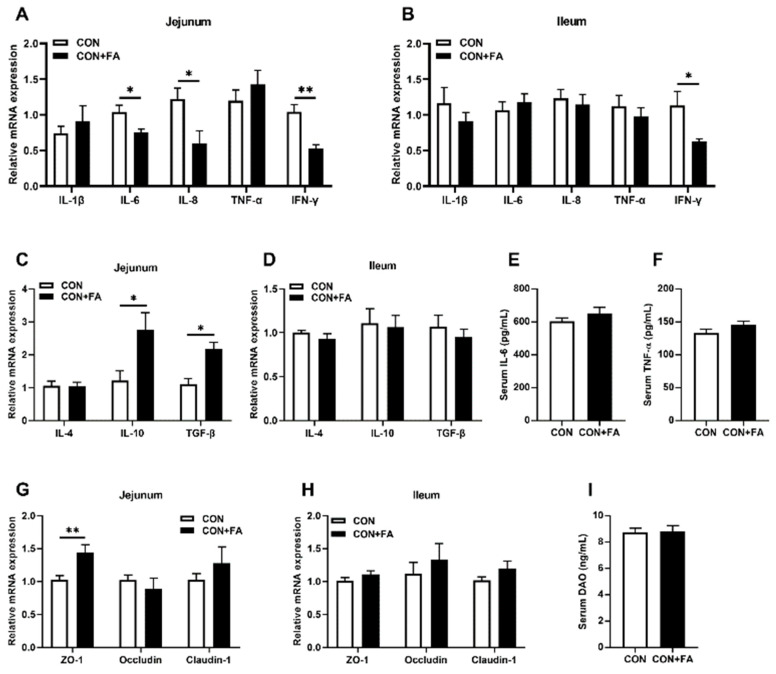
Maternal folic acid supplementation slightly improves intestinal inflammatory response in offspring. (**A**,**B**) Relative mRNA expression levels of proinflammatory cytokines, *IL-1β*, *IL-6*, *IL-8*, *TNF-α*, and *IFN-γ*, in the jejunum and ileum. (**C**,**D**) Relative mRNA expression levels of anti-inflammatory cytokines, *IL-4*, *IL-10*, and *TGF-β*, in the jejunum and ileum. (**E**) Serum IL-6 level. (**F**) Serum TNF-α level. (**G**,**H**) Relative mRNA expression levels of intestinal tight junction proteins, *ZO-1*, *Occludin*, and *Claudin-1*, in the jejunum and ileum. Jejunal gene mRNA expression. (**I**) Serum DAO level. The mRNA expression was normalized to *β-actin*. The data are shown as means ± SEM of three independent tests. Tukey’s *t*-test between two groups and one-way ANOVA among multiple groups were used to generate *p* values. * *p* < 0.05, ** *p* < 0.01 indicate a significant difference (*n* = 5).

Weaning stress activates the intestinal immune system and causes a flood of pro-inflammatory cytokines to be produced, resulting in intestinal damage and malfunction [14]. We therefore detected inflammatory cytokines to evaluate the role of maternal folic acid supplementation in terms of intestinal inflammatory in offspring. Compared with weaning piglets born to dams in the CON group, the mRNA levels of pro-inflammatory cytokines, such as interleukin *(IL)-6*, *IL-8*, and interferon-γ (*IFN-γ*), were all significantly (*p* < 0.05) decreased in the jejunum of piglets born to dams in the CON + FA group (Figure 4A). *IFN-γ* mRNA expression was significantly (*p* < 0.05) lower in the ileum of piglets born to dams with folic acid supplementation (Figure 4B). In contrast, the transcripts of anti-inflammatory cytokines including *IL-10* and transforming growth factor-β (*TGF-β*) were both increased significantly (*p* < 0.05) in the jejunum of weaning piglets born to dams in the CON + FA group compared with those in the CON group (Figure 4C), while they were no changes in the ileum (Figure 4D). Meanwhile, there was no significant difference in serum levels of IL-6 and tumor necrosis factor-α (TNF-α) (Figure 4E,F). Furthermore, the mRNA level of the tight junction protein *ZO-1* was significantly (*p* < 0.01) increased in the jejunum of weaning piglets born to dams with folic acid supplementation (Figure 4G), while those of *Occludin* and *Claudin-1*, the other two main tight junction markers, showed no significant difference (Figure 4G,H). There was still no significant change in the serum diamine oxidase (DAO) level (Figure 4I). The above results indicated that maternal folic acid supplementation could not significantly improve intestinal integrity, but did slightly improve the intestinal inflammatory response in offspring during weaning stress. 

### 3.5. Maternal Folic Acid Supplementation Stimulates the Proliferation of Intestinal Cells in Offspring

**Figure 5:**
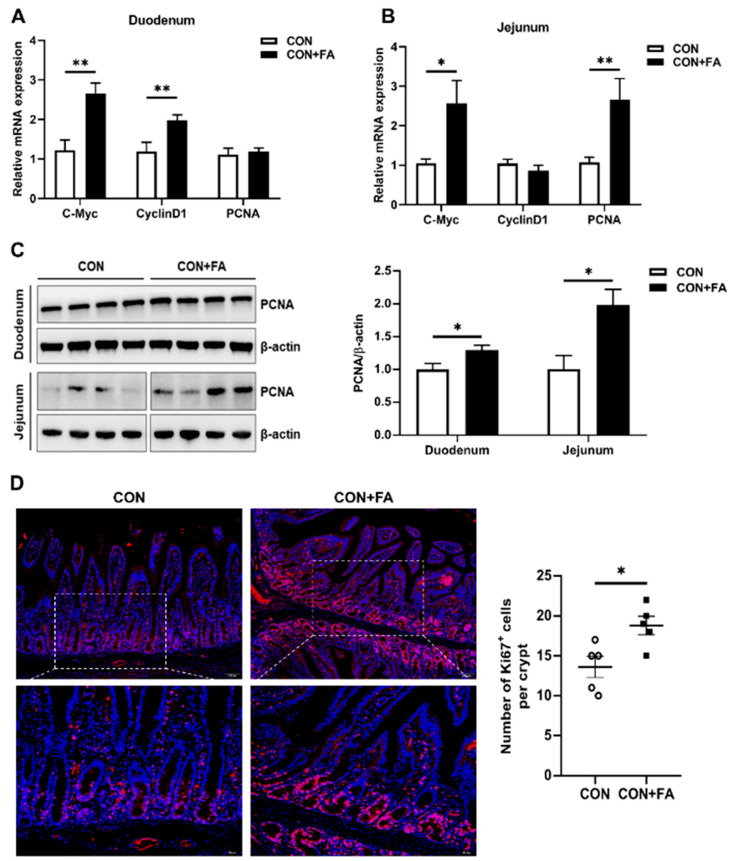
Maternal folic acid supplementation stimulates the proliferation of intestinal cells in offspring. (**A**,**B**) Relative mRNA expression levels of proliferation-related markers, *C-Myc*, *cyclinD1*, and *PCNA*, in the duodenum and jejunum. (**C**) Relative protein expression of PCNA in the duodenum and jejunum. The mRNA and protein expression were both normalized to *β-actin*. (**D**) Representative images of immunofluorescence of Ki67 cells and quantification of positive cells in each crypt using the Image J software (V1.8.0.112). Fluorescence microscopy was used to detect the expression of the Ki67 gene (red) in cells. DAPI (blue) was used to mark cell nuclei. For the detection of fluorescence intensity, the number of cells was quantified to the same level. The data are shown as means ± SEM of three independent tests. Tukey’s *t*-test between two groups and one-way ANOVA among multiple groups were used to generate *p* values. * *p* < 0.05, ** *p* < 0.01 indicate a significant difference (*n* = 5).

In order to explore the role of maternal folic acid supplementation in terms of the proliferation of intestinal cells in offspring, we examined the gene expression of proliferation-related markers in the piglets. The transcripts of *C-Myc* and *CyclinD1* were both significantly (*p* < 0.01) increased in the duodenum of piglet born to dams with folic acid supplementation (Figure 5A). And in the jejunum, the mRNA levels of *C-Myc* and *PCNA* were significantly (*p* < 0.05) higher in the piglets with folic acid supplementation compared with those in control piglets (Figure 5B). Western blot analysis confirmed that the protein levels of PCNA were significantly (*p* < 0.05) increased both in the duodenum and jejunum of the folic-acid-supplemented piglets compared with the control piglets (Figure 5C). Moreover, intestinal stem cells (ISCs) are highly proliferating cells that fuel the ongoing regeneration of the intestinal epithelium. We measured the number of proliferation cells in ISCs of the jejunal crypt of piglets. Immunofluorescence results showed that the number of Ki67-positive cells was significantly (*p* < 0.05) increased by 38% in the jejunum of piglets with folic acid supplementation compared with those in control piglets (Figure 5D). These results indicated that maternal folic acid supplementation could stimulate the proliferation of intestinal cells and ISCs in offspring.

### 3.6. Maternal Folic Acid Supplementation Promotes the Differentiation of Intestinal Stem Cells in Offspring

**Figure 6:**
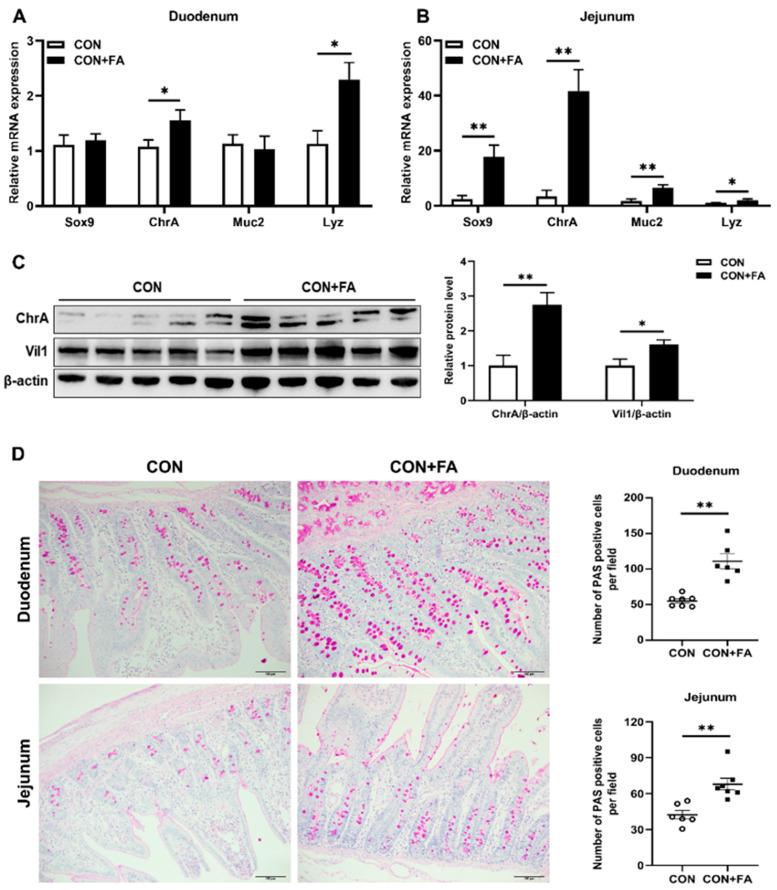
Maternal folic acid supplementation promotes the differentiation of intestinal stem cells in offspring. (**A**,**B**) Relative mRNA expression levels of secreted mature cells markers, *Sox9*, *ChrA*, *Muc2*, and *Lyz*, in the duodenum and jejunum. (**C**) Relative protein expression of epithelial cell markers ChrA and Vil1 in the jejunum. The mRNA and protein expression were both normalized to *β-actin*. (**D**) Representative images of goblet cells and quantification of positive cells in five random fields of view/each section using the Image J software. Purple: neutral mucin, Light blue: acidic mucin. The data are shown as means ± SEM of three independent tests. Tukey’s *t*-test between two groups and one-way ANOVA among multiple groups were used to generate *p* values. * *p* < 0.05, ** *p* < 0.01 indicate a significant difference (*n* = 5).

In order to determine if the differentiation of intestinal stem cells of offspring was related to folic acid supplementation, we measured the expression of marker genes of ISCs (Sox9) and secreted mature cells derived from ISCs (ChrA, Muc2 and Lyz) in the duodenum and jejunum of piglets. Compared with weaned piglets born to dams in the CON group, the mRNA levels of *ChrA* and *Lyz* were both significantly (*p* < 0.05) increased in the duodenum of weaned piglets born to dams in the CON + FA group (Figure 6A). And in the jejunum, the gene expressions of *Sox9*, *ChrA*, *Muc2*, and *Lyz* were all significantly (*p* < 0.05) higher in piglets born to dams with folic acid supplementation than those born to control dams (Figure 6B). Furthermore, Western blot analysis confirmed that the protein levels of ChrA and Vil1, an absorptive mature cell marker gene, were significantly (*p* < 0.05) increased in the jejunum of folic-acid-supplemented piglets compared with control piglets (Figure 6C). In addition, PAS staining showed that the number of PAS-positive cells in both the duodenum and jejunum of folic-acid-supplemented piglets were significantly (*p* < 0.01) increased compared with the control piglets (Figure 6D). The above results indicated that maternal folic acid supplementation promoted the differentiation of ISCs in offspring, which is beneficial to intestinal development and absorption.

## 4. Discussion

Folic acid is an essential vitamin for humans and plays a crucial role in early embryonic development. It is recommended to supplement with folic acid 5–6 months prior to conception as the optimal approach to reduce the risk of neural tube defects in newborns [4,18]. Research has demonstrated that folic acid supplementation has a positive impact on gut health by improving the diversity of gut microbiota and length of intestinal villi of weaned piglets [19]. Maternal folic acid supplementation also plays fundamental roles in the programming of offspring gut health, which significantly reduces the odds of colorectal adenocarcinoma by 64% in the offspring [6]. However, the mechanisms through which folic acid influences offspring gut development and health remain poorly understood. Here, we found that maternal folic acid supplementation contributes to the intestinal health of the offspring by promoting the proliferation of intestinal stem cells. To our knowledge, this is the first study to focus on the effects of maternal folic acid supplementation during pregnancy on the intestinal development of offspring.

Our study revealed that maternal folic acid supplementation could elevate serum folate levels of the offspring, which is consistent with previous research [6,20]. There are three specific folic acid transporters in the intestine, FOLR, RFC, and PCFT [21]. FOLRs are very high-affinity folate-binding proteins that facilitate folate transportation via receptor-mediated endocytosis [22]. Liu et al. reported that folic acid perfusion improved FOLR expression [23], corroborating our current findings. At the same time, we found that the mRNA expression of *DHFR* and *MTHFR* was significantly increased in the intestines of piglets belonging to the CON + FA group, compared with those in the CON group. DHFR and MTHFR are recognized as crucial enzymes involved in intracellular folate metabolism [24]. A previous study showed that dietary folic acid supplementation leads to an augmented abundance of *DHFR* and *MTHFR* mRNA in tissues, however, folate over-supplementation has been shown to inactivate methionine synthase (MS) and MTHFR expression [25]. Thus, it is hypothesized that a moderate level of maternal folic acid supplementation promotes absorption and transportation of folate in the offspring’s intestines. Consequently, key enzymes involved in folate metabolism have appropriate substrate concentrations, making enzyme-catalyzed reactions more active.

Weaning stress has a significant detrimental impact on the integrity of intestinal epithelial cells, resulting in atrophy of intestinal villi and hyperplasia of crypts in piglets [26]. The ratio of villus height to crypt depth can serve as an indicator of the small intestine’s capacity for nutrient digestion and absorption [12]. In our study, we observed a significant increase in the ratio of villus height to crypt depth in the duodenum and jejunum in weaning pigs due to maternal folic acid supplementation. The migration of intestinal epithelial cells along the crypt–villus axis is accompanied by their proliferation and differentiation, as previously noted [27]. As a result, a higher ratio of villus height to crypt depth may suggest increased proliferation and differentiation. It is reasonable to believe that maternal folic acid supplementation may improve offspring’s digestive and absorptive capabilities by stimulating the proliferation and differentiation of intestinal epithelial cells.

The development of the small intestine’s absorption function was significantly reliant on intestinal nutrient transporters [28]. Here, we examined the transcription levels of the glucose transporters *SGLT1* and *GLUT4*, amino acid transporters *SNAT2*, *ASCT2*, and *SLC7A5*, as well as the fatty acid transporter *FABP2* in the duodenum and jejunum of piglets. These transporters were found to be expressed by enterocytes, a specialized type of mature absorptive cells. Enterocytes employ these transport channels to facilitate the absorption of various nutrients, including glucose, lactose, glutamate, and long-chain fatty acids. Generally, the increased expression of nutrient transporters is associated with higher intestinal absorption. In the current study, we also found a significant increase in the mRNA levels of *FABP2*, which is involved in the transport of fatty acids, in the piglets’ duodenum and jejunum of the CON + FA group. FABP2 is specifically expressed in enterocytes of the small intestine epithelium, which was also used as a marker for enterocytes [29]. Thus, the significant upregulation of *FABP2* expression might indicate a substantial augmentation of the number of enterocytes within the duodenum and jejunum of piglets in the CON + FA group. 

While previous studies have documented the impact of folic acid on the proliferation and differentiation of pancreatic stem cells [30], C2C12 myoblasts [31], mesenchymal stem cells [32], and colorectal cancer cells [33], little is known on whether folic acid affects the differentiation of ISCs into enterocytes. We found an involvement of folic acid in the proliferation of ISCs and their differentiation into enterocytes, based on the presence of similar biological properties among stem cells. Interestingly, our findings discovered that the administration of folic acid resulted in the promotion of the differentiation of ISCs into intestinal cells. Additionally, we observed a significant increase in the expression of proliferation-related marker genes, namely, *C-Myc*, *CyclinD1*, and *PCNA*, as well as an augmented number of cells in the crypt proliferating area within the duodenum and jejunum of piglets. These findings collectively suggest that the proliferative capacity of ISCs was also enhanced during this process. As a result, the potential of folic acid to enhance differentiation of ISCs could be viewed as an accumulative influence, thereby providing a plausible explanation for why folic acid promotes both ISC proliferation and differentiation. In addition, we found that maternal folic acid supplementation not only promoted the differentiation of absorptive mature intestinal cells (enterocytes), but also significantly upregulated the number of secretory mature intestinal cells, including goblet cells, paneth cells, and enteroendocrine cells. Both mucins and cytokines that are secreted by secretory mature intestinal cells are important components of intestinal barriers [34]. These results indicated that folic acid may not only promote the differentiation of ISCs into enterocytes, thus promoting intestinal absorption capacity, but also positively influences the development and formation of intestinal barriers. Our results support this hypothesis, as maternal folic acid supplementation was found to modestly ameliorate the intestinal inflammatory response in the offspring. However, the precise mechanism remains unclear. Further investigation could center on elucidating the impact of folic acid on the differentiation of secretory cells and on protecting the intestinal development. It has been found that WNT/β-catenin is an important pathway protein molecule in the proliferation of ISCs and intestinal development [35], and its role may be related to DNA synthesis and methylation modification, which were connected to folic acid [36]. However, additional experimental research is required to ascertain the specific mechanism.

## 5. Conclusions

We first found that maternal folic acid supplementation could enhance the growth performance and intestinal health of offspring piglets by promoting the equilibrium of epithelial cell renewal. Maternal folic acid supplementation could promote the proliferation and differentiation of ISCs in the offspring, which might enhance the intestinal digestion and absorption function.

## Figures and Tables

**Table 1:** Formulation and proximate composition of the basal diets (as-fed basis, %).

Item	Content
**Ingredients**	
Corn	64.70
Soybean meal	7.00
Wheat bran	24.00
Dicalcium phosphate	0.80
Limestone	1.50
Salt	0.40
Lysine monohydrochloride, 98.5%	0.11
Phytase	0.01
Choline chloride, 60%	0.10
Yeast powder	0.05
Potassium magnesium sulfate	0.20
Premix ^1^	1.13
Total	100.00
**Nutrient levels**	
Crude protein	≥13.50
Crude ash	≤10.00
Crude fiber	≤10.00
Lysine	≥0.60
Calcium	1.20
Total phosphorus	≥0.05

^1^ The premix provided the following per kilogram of diet: vitamin A, 10 kIU; vitamin D3, 1 kIU; vitamin E, 60 IU; vitamin B1, 2 mg; vitamin B2, 4 mg; vitamin B6, 3 mg; vitamin B12, 30 µg; niacin, 3 mg; folic acid,1.30 mg; copper, 15 mg; iron, 110 mg; zinc, 100 mg; manganese 20 mg; iodine, 0.2 mg; and selenium, 0.3 mg.

**Table 2:** Forward and reverse primer sequences for PCR analysis.

Gene	Primer Sequences (5′ to 3′)		Size (bp)
β-actin	F: CGAGACCTTCAACACCCCAG	R: AGTCCATCACGATGCCAGTG	92
FOLR1	F: TCTATGAGTGCTCGCCCAACC	R: GGCAGTCTTCCCACCAGTTCT	123
DHFR	F: TCCCAGAACTTGGGCATTGG	R: CCAGGTCTTCCTACCCATAATCA	141
MTHFR	F: AAGCGTCGGGAGGAAGATGTC	R: CAAAGGCGGGAGAGGAGGAG	127
MTRR	F: ACAAGTACGATCTGAGGACGGAAG	R: CGCAGGTGAGCAAGGAGGTC	145
SGLT1	F: AGTGGGCAGCTCTTCGATTA	R: CCAGCCCAATCATACATCCT	148
GLUT4	F: TCATCATCGGCATGAGTTTC	R: CGGGTTTCAGGCACTTTTAG	121
SNAT2	F: TACTTGGTTCTGCTGGTGTCC	R: GTTGTGGGCTGTGTAAAGGTG	212
ASCT2	F: GGATTCTGGACCGCTGCCTT	R: GGCTCCTCCGCTCTTCGTTT	362
FABP2	F: GCCTGGAAGATAGACCGCAATGAG	R: AGTTCCGTCTGCGAGGCTGTAG	216
IL-1β	F: ATTCAGGGACCCTACCCTCTC	R: CTTCTCCACTGCCACGATGA	279
IL-6	F: ACAAAGCCACCACCCCTAAC	R: CGTGGACGGCATCAATCTCA	185
IL-8	F: GCAAGAGTAAGTGCAGAACTTCG	R: GGGTGGAAAGGTGTGGAATG	62
TNF-α	F: TAAGGGCTGCCTTGGTTCAG	R: AGAGGTTCAGCGATGTAGCG	186
IFN-γ	F: CGCAAAGCCATCAGTGAACTCATC	R: TTTGATGCTCTCTGGCCTTGGAAC	110
IL-4	F: CAGCTTCAACACTTTGAGTATTTC	R: GCGACATCACCTTACAAGAGAT	328
IL-10	F: TAATGCCGAAGGCAGAGAGT	R: GGCCTTGCTCTTGTTTTCAC	134
TGF-β	F: GGACCTTATCCTGAATGCCTT	R: TAGGTTACCACTGAGCCACAAT	133
ZO-1	F: GCATGATGATCGTCTGTCCTACC	R: CCGCCTTCTGTATCTGTGTCTTC	108
Occludin	F: ACTTCAGATCAACAAAGGCAAC	R: CCAGCTCTTTATCCAGTCGAGA	124
Claudin-1	F: AGAAATTTGTTGATCCCGGAAACCA	R: TAAGCTCCGGCAAATACAAGCA	152
C-Myc	F: GAACCCTTGGCTCTCCACGA	R: GCTGTGAGGAGGTTTGCTGT	174
CyclinD1	F: GTGAAAAAGAGCCGCCTGC	R: CGGATGGAGTTGTCGGTGTAG	119
PCNA	F: GCAAGTGGAGAACTCGGAAATG	R: GTAGGAGAGAGTGGAGTGGCT	161
Sox9	F: GCGGAGAAAGTCGGTGAAGAAC	R: AGATGGCGTTGGGAGAGATGTG	80
Muc2	F: GGCTGCTCATTGAGAGGAGT	R: ATGTTCCCGAACTCCAAGG	214
ChrA	F: GACCTCGCTCTCCAAGGAGCCA	R: TGTGCGCCTGGGCGTTTCTT	332
Lyz	F: GGTCTATGATCGGTGCGAGT	R: AACTGCTTTGGGTGTCTTGC	214

FOLR1 = folate receptor 1; DHFR = dihydrofolate reductase; MTHFR = methylenetetrahydrofolate reductase; MTRR = 5-methyltetrahydrofolate-homocysteine methyltransferase reductase; SGLT1 = solute carrier family 5 member 1; GLUT4 = solute carrier family 2 member 4; SNAT2 = solute carrier family 38 member 2; ASCT2 = solute carrier family 1 member 5; FABP2 = fatty-acid-binding protein 2; IL = interleukin; TNF = tumor necrosis factor; IFN = interferon; TGF = transforming growth factor; ZO-1 = zonula occludens 1; C-Myc = MYC proto-oncogene; PCNA = proliferating cell nuclear antigen; Sox9 = SRY-box 9; Muc2 = mucin 2; ChrA = chromogranin A; Lyz = lysozyme.

## Data Availability

The data used to support the findings of this study are available from the corresponding author upon request.

## References

[1] Li Q., Yang S., Zhang X., Liu X., Wu Z., Qi Y., Guan W., Ren M., Zhang S. (2022). Maternal nutrition during late gestation and lactation: Association with immunity and the inflammatory response in the offspring. Front. Immunol..

[2] Levine S.Z., Kodesh A., Viktorin A., Smith L., Uher R., Reichenberg A., Sandin S. (2018). Association of maternal use of folic acid and multivitamin supplements in the periods before and during pregnancy with the risk of autism spectrum disorder in offspring. JAMA Psychiatry.

[3] Wang B., Li H., Li Z., Jian L., Gao Y., Qu Y., Liu C., Xu C., Li Y., Diao Z. (2019). Maternal folic acid supplementation modulates the growth performance, muscle development and immunity of Hu sheep offspring of different litter size. J. Nutr. Biochem..

[4] van Gool J.D., Hirche H., Lax H., Schaepdrijver L.D. (2018). Folic acid and primary prevention of neural tube defects: A review. Reprod. Toxicol..

[5] Shulpekova Y., Nechaev V., Kardasheva S., Sedova A., Kurbatova A., Bueverova E., Kopylov A., Malsagova K., Dlamini J.C., Ivashkin V. (2021). The concept of folic acid in health and disease. Molecules.

[6] Sie K.K., Medline A., van Weel J., Sohn K.J., Choi S.W., Croxford R., Kim Y.I. (2011). Effect of maternal and postweaning folic acid supplementation on colorectal cancer risk in the offspring. Gut.

[7] He Q., Zou T., Chen J., Jian L., He J., Xia Y., Xie F., Wang Z., You J. (2022). Maternal methyl-donor micronutrient supplementation during pregnancy promotes skeletal muscle differentiation and maturity in newborn and weaning pigs. Front. Nutr..

[8] Engevik M.A., Morra C.N., Röth D., Engevik K., Spinler J.K., Devaraj S., Crawford S.E., Estes M.K., Kalkum M., Versalovic J. (2019). Microbial metabolic capacity for intestinal folate production and modulation of host folate receptors. Front. Microbiol..

[9] Czeizel A.E., Dudás I. (1992). Prevention of the first occurrence of neural-tube defects by periconceptional vitamin supplementation. N. Engl. J. Med..

[10] Kancherla V. (2023). Neural tube defects: A review of global prevalence, causes, and primary prevention. Child’s Nerv. Syst..

[11] Bibbins-Domingo K., Grossman D.C., Curry S.J., Davidson K.W., Epling J.W., García F.A., Kemper A.R., Krist A.H., Kurth A.E., US Preventive Services Task Force (2017). Folic acid supplementation for the prevention of neural tube defects: US preventive services task force recommendation statement. JAMA.

[12] Liu H., Wang J., Mou D., Che L., Fang Z., Feng B., Lin Y., Xu S., Wu D. (2017). Maternal methyl donor supplementation during gestation counteracts the bisphenol a-induced impairment of intestinal morphology, disaccharidase activity, and nutrient transporters gene expression in newborn and weaning pigs. Nutrients.

[13] Yang H., Xiong X., Wang X., Tan B., Li T., Yin Y. (2016). Effects of weaning on intestinal upper villus epithelial cells of piglets. PLoS ONE.

[14] Wang L., Tan X., Wang H., Wang Q., Huang P., Li Y., Li J., Huang J., Yang H., Yin Y. (2021). Effects of varying dietary folic acid during weaning stress of piglets. Anim. Nutr..

[15] Catala G.N., Bestwick C.S., Russell W.R., Tortora K., Giovannelli L., Moyer M.P., Lendoiro E., Duthie S.J. (2019). Folate, genomic stability and colon cancer: The use of single cell gel electrophoresis in assessing the impact of folate in vitro, in vivo and in human biomonitoring. Mutat. Res. Genet. Toxicol. Environ. Mutagen..

[16] Ichi S., Nakazaki H., Boshnjaku V., Singh R.M., Mania-Farnell B., Xi G., McLone D.G., Tomita T., Mayanil C.S. (2012). Fetal neural tube stem cells from Pax3 mutant mice proliferate, differentiate, and form synaptic connections when stimulated with folic acid. Stem Cells Dev..

[17] Wei T., Jia W., Qian Z., Zhao L., Yu Y., Li L., Wang C., Liu Q., Yang D., Wang G. (2017). Folic acid supports pluripotency and reprogramming by regulating LIF/STAT3 and MAPK/ERK signaling. Stem Cells Dev..

[18] Ferrazzi E., Tiso G., Martino D.D. (2020). Folic acid versus 5-methyl tetrahydrofolate supplementation in pregnancy. Eur. J. Obstet. Gynecol. Reprod. Biol..

[19] Wang L., Zou L., Li J., Yang H., Yin Y. (2021). Effect of dietary folate level on organ weight, digesta pH, short-chain fatty acid concentration, and intestinal microbiota of weaned piglets. J. Anim. Sci..

[20] Meher A., Joshi A., Joshi S. (2014). Differential regulation of hepatic transcription factors in the Wistar rat offspring born to dams fed folic acid, vitamin B12 deficient diets and supplemented with omega-3 fatty acids. PLoS ONE.

[21] Zhao R., Matherly L.H., Goldman I.D. (2009). Membrane transporters and folate homeostasis: Intestinal absorption and transport into systemic compartments and tissues. Expert Rev. Mol. Med..

[22] Salazar M.D., Ratnam M. (2007). The folate receptor: What does it promise in tissue-targeted therapeutics?. Cancer Metastasis Rev..

[23] Liu Y., Liu X., Zhou J., Ren Z., Yang X., Cao Y., Yang X. (2019). Folic acid perfusion administration reduced abdominal fat deposition in starter Arbor Acres broilers. Poult. Sci..

[24] Bhatia M., Thakur J., Suyal S., Oniel R., Chakraborty R., Pradhan S., Sharma M., Sengupta S., Laxman S., Masakapalli S.K. (2020). Allosteric inhibition of MTHFR prevents futile SAM cycling and maintains nucleotide pools in one-carbon metabolism. J. Biol. Chem..

[25] Ortbauer M., Ripper D., Fuhrmann T., Lassi M., Auernigg-Haselmaier S., Stiegler C., König J. (2016). Folate deficiency and over-supplementation causes impaired folate metabolism: Regulation and adaptation mechanisms in Caenorhabditis elegans. Mol. Nutr. Food Res..

[26] Hu C.H., Xiao K., Luan Z.S., Song J. (2013). Early weaning increases intestinal permeability, alters expression of cytokine and tight junction proteins, and activates mitogen-activated protein kinases in pigs. J. Anim. Sci..

[27] Mariadason J.M., Nicholas C., L’Italien K.E., Zhuang M., Smartt H.J., Heerdt B.G., Yang W., Corner G.A., Wilson A.J., Klampfer L. (2005). Gene expression profiling of intestinal epithelial cell maturation along the crypt-villus axis. Gastroenterology.

[28] Mickiewicz M., Zabielski R., Grenier B., Normand L.L., Savary G., Holst J.J., Oswald I.P., Metges C.C., Guilloteau P. (2012). Structural and functional development of small intestine in intrauterine growth retarded porcine offspring born to gilts fed diets with differing protein ratios throughout pregnancy. J. Physiol. Pharmacol..

[29] Gjorevski N., Nikolaev M., Brown T.E., Mitrofanova O., Brandenberg N., DelRio F.W., Yavitt F.M., Liberali P., Anseth K.S., Lutolf M.P. (2022). Tissue geometry drives deterministic organoid patterning. Science.

[30] Yang H., Qin D., Xu S., He C., Sun J., Hua J., Peng S. (2021). Folic acid promotes proliferation and differentiation of porcine pancreatic stem cells into insulin-secreting cells through canonical Wnt and ERK signaling pathway. J. Steroid Biochem. Mol. Biol..

[31] Hwang S.Y., Kang Y.J., Sung B., Jang J.Y., Hwang N.L., Oh H.J., Ahn Y.R., Kim H.J., Shin J.H., Yoo M.A. (2018). Folic acid is necessary for proliferation and differentiation of C2C12 myoblasts. J. Cell. Physiol..

[32] Moon Y., Patel M., Um S., Lee H.J., Park S., Park S.B., Cha S.S., Jeong B. (2022). Folic acid pretreatment and its sustained delivery for chondrogenic differentiation of MSCs. J. Control Release.

[33] Ting P.C., Lee W.R., Huo Y.N., Hsu S.P., Lee W.S. (2019). Folic acid inhibits colorectal cancer cell migration. J. Nutr. Biochem..

[34] Turner J.R. (2009). Intestinal mucosal barrier function in health and disease. Nat. Rev. Immunol..

[35] Zhou J.Y., Huang D.G., Zhu M., Gao C.Q., Yan H.C., Li X.G., Wang X.Q. (2020). Wnt/β-catenin-mediated heat exposure inhibits intestinal epithelial cell proliferation and stem cell expansion through endoplasmic reticulum stress. J. Cell. Physiol..

[36] Crott J.W., Liu Z., Keyes M.K., Choi S.W., Jang H., Moyer M.P., Mason J.B. (2008). Moderate folate depletion modulates the expression of selected genes involved in cell cycle, intracellular signaling and folate uptake in human colonic epithelial cell lines. J. Nutr. Biochem..

